# Academic misconduct among students in medical colleges of Karachi, Pakistan

**DOI:** 10.12669/pjms.293.3300

**Published:** 2013

**Authors:** Kamran Hafeez, Muhammad Laiq-uz-Zaman Khan, Masood Jawaid, Saroona Haroon

**Affiliations:** 1 Dr. Kamran Hafeez, FCPS, Assistant Professor Orthopedics, Dow International Medical College, Karachi, Pakistan.; 2 Dr. Muhammad Laiq-uz-Zaman Khan, FCPS, Assistant Professor Surgery, Dow International Medical College, Dow University Hospital, Ojha Campus, KDA Scheme 33, Gulzar-e-Hijri, Suparco Road, Karachi, Pakistan.; 3 Dr. Masood Jawaid, FCPS, Assistant Professor Surgery, Dow International Medical College, Dow University Hospital, Ojha Campus, KDA Scheme 33, Gulzar-e-Hijri, Suparco Road, Karachi, Pakistan.; 4 Dr. Saroona Haroon, FCPS, Senior Instructor, Histopathology, Department of Pathology, Aga Khan University Hospital, Karachi, Pakistan.

**Keywords:** Academic misconduct, Medical students

## Abstract

***Objective:*** To determine the trends of academic misconduct in undergraduate students of different private and government section medical institutes.

***Methodology:*** This cross sectional study was conducted at three medical colleges of Karachi, Pakistan. The students were evaluated by giving a self reported questionnaire containing various questions assessing their educational dishonesty and cheating behaviors.

***Results:*** A total of 274 students from different years completed the questionnaire. Mean age was 21.48 ± 1.89 years. Most of the students were in 4^th^ year (n=86; 31.3%). There were 182 (66.5%) females and 92 (33.5%) males. Majority of the students (n=155; 55.1%) accepted that they have cheated at least once. There was no significant difference regarding acceptance of cheating among different years of study (p=0.23) however females were found to accept cheating more as compared to males (p=0.036). First year students were found more to ask teachers for answers during OSCE (p=0.01). A large number of students accepted that they mark proxy for their friends (85.7%) and also ask their friends to mark proxy for them (85.03%). Nearly half (44.02%) of the students rotating in wards also admitted to write fake histories.

***Conclusion:*** A large number of medical students admitted cheating and involvement in other academic misconduct. We need to improve our educational system, formally add professional session and strict disciplinary action should be taken against those who are found guilty.

## INTRODUCTION

Academic misconduct is defined as any action or attempted action that may result in creating an unfair academic advantage for oneself or an unfair academic advantage or disadvantage for any other member or members of the academic community.^1^ It is an unacceptable mean to achieve a higher grade. Good medical professional in addition to knowledge and skills must be equipped with high ethical and moral standards.^2^^,^^3^ Medical students are reported to be involved in academic misconduct. In one study from Croatia, Hrabak et al reported 94% of students admitted cheating at least once in their college life.^4^ In another study Baldwin et al reported that 39% of students witnessed some form of cheating among their colleagues in medical schools while only 4.7% admitted themselves to be involved in cheating during their medical school.^5^ It may lead to poor knowledge which may be harmful to the patients.^6^ High prevalence of academic misconduct among medical students can affect their professionalism in future career.^7^ Professionalism is an important aspect of medical career. Unprofessional practitioners can show shirking responsibility, manipulate medical records, hospitalize patients for better reimbursements, fabricate clinical data for research etc.^8^ In general academic misconduct is not acceptable. Generally students also consider it immoral and prefer not to be involved in it.^9^ One of the recent studies; reported academic misconduct including cheating as an acceptable behavior among medical students.^10^ Number of factors has been proposed as the causal factors for academic misconduct including age, gender, personality, institutional factors, competitive pressure and stress.^11^^,^^12^

A graduating student must be an honest medical professional. Students should be taught about the fundamentals of professionalism in the undergraduate curricula and trained to avoid academic dishonesty. Academic misconduct might be a bigger problem in our country also. The objective of this study was to determine the trend of academic misconduct in our medical undergraduate students.

## METHODOLOGY

This cross sectional study was conducted at three medical colleges in Karachi, Pakistan during September 2011. Students from different medical colleges were approached and questionnaires were distributed in the classrooms. Time was allocated at the end of lecture in a specified classroom and students were briefed about the study. Students were asked to fill the required questionnaire voluntarily after consent. Students were assured that they can withdraw at anytime without reprisal and their anonymity will be maintained. First part of the questionnaire contained general information regarding their demographics and year of study. Second part consisted of fifteen survey questions (as shown in Table-I) assessing the behaviors of medical students regarding cheating and academic misconduct.

The data was analyzed using SPSS (SPSS Inc., Chicago, Illinois, USA). Demographics were presented as descriptive statistics while Chi-square test was used to compare between groups. P value <0.05 was considered significant.

## RESULTS

A total of 274 students from different years completed the questionnaire, representing 98(35.8%), 90(32.97%) and 86(31.4%) students from three medical colleges respectively. Mean age was 21.48 ± 1.89 years. Most of the students were in 4^th^ year (n=86; 31.3%) followed by 59 (21.5%) in final year, 46 (16.8%) in 2^nd^ year, 44 (16.12%) in 1^st^ year and 39 (14.2%) in 3^rd^ year. There were 182 (66.5%) females and 92 (33.5%) males. Majority of the students (n=155; 55.1%) accepted that they have cheated at least once. There was no significant difference regarding acceptance of cheating among different years of study (p=0.23), however, females accepted to have been more involved in cheating as compared to males (p=0.036).

First year students were found more to ask teachers for answers during OSCE (p=0.01). A large number of students accepted that they mark proxy for their friends (85.7%) and also ask their friends to mark proxy for them (85.03%). Few students also accepted to forge teacher’s signature (16.9%). Nearly half of the students rotating in wards also admitted to write fake histories (44.02%). Behaviours of medical students regarding academic misconduct are presented in Table-I.

## DISCUSSION

In our study we found that majority of students (55.1%) cheated at least once in their medical college. Female students accepted to have cheated more as compared to male students. A large number of students accepted to mark proxy for their friends (85.7%). Some of the students also accepted to have forged a teacher’s signature (16.9%). The attitude towards cheating reflects the moral and cultural values of a society in which the child is brought up. We can not expect a medical student to behave in a different way as compared to other students in the same community.^13^ Although there are instructions available over examination copies as well as available in institutional policies to keep students away from these unlawful acts but still students get involved in it. This reflect that they may be unaware of the consequences and it may be a contributory factor in increasing trend toward academic misconduct. Students get involved in this behavior to achieve good grades but are not aware of its long term hazards which may affect their future professional development; and the punishment which they may face against this unlawful activity.^14^^,^^15^ Irresponsible attitudes at undergraduate level may affect professional growth in the careers of future health care professionals.^16^

There was no major difference in most of the scenarios however first year medical students were found more to ask teachers for answers. Baldwin et al, showed that majority of the medical students admitted cheating in high school and junior high school before coming to medical school.^5^ This emphasizes that steps should be taken at the time of admission process and students should be educated regarding institutional policies about academic misconduct.

**Table-I:** Behaviors of medical students regarding academic misconduct (n=274).

*Questions*	*Yes* *n (%)*
Have you ever cheated during any exam?	151 (55.1)
How often do you cheat during exams?	
Always Often Sometimes	15 (5.5) 28 (10.2) 107 (39.1)
Have you used mobile phone to exchange answers during an exam?	19 (6.9)
Do you try to find out about test questions in advance?	
Always Often Sometimes	48 (17.5) 20 (7.3) 97 (35.4)
Have you ever marked answers on the question paper during the OSCE/OSPE?	117 (42.7)
Do you ask the teacher for answers during OSCE?	50 (18.2)
Do you tell your friends the questions which were asked in first shift in the OSCE?	229 (83.6)
Have you ever copied assignments/presentations from your seniors/class mates?	
Always Often Sometimes	32 (11.7) 28 (10.2) 92 (33.6)
Why do you prefer copying assignments/presentations?	
Its useless exercise Its waste of time I don’t have time I don’t learn anything	35 (12.8) 59 (21.5) 70 (25.5) 17 (6.2)
Do you mark proxy for your friends?	235(85.7)
Have you ever asked your friend to mark your attendance?	233(85.03)
Have you ever forged a teacher signature?	46(16.9)
Have you ever paid anyone to pass an exam?	14(5.1)
	
*Academic misconduct in 3* ^rd^ *, 4* ^th^ * and final year: (n=184)*	
Do you write fake histories for assignments?	81(44.02)
Do you write fake examination findings without performing it?	53(28.8)

In this study female students admitted cheating more as compared to male students. Gender difference is also reported vastly in literature. In some studies men were reported to cheat more ^11^^,^^15^ while in another study there was no significant difference between genders.^17^

Hrabak et al^4^, reported his results of 827 medical students and found that 94% were involved in cheating. The most common act of misconduct was to sign a proxy for an absent friend. In our study 55.1% of the students admitted cheating and similarly the most frequent act of misconduct was to sign a proxy for an absent friend (85.7%).

A large number of students accepted copying assignments from their senior class mates. Most of them think it as a waste of time. This reflects the lack of interest of students in the assignments. There is need to improve our curricula and make assignments interesting so that students take active part in this activity.^18^

Easy access to cell phones nowadays can be a threat to increase in cheating in class rooms. Students are becoming experts in text messaging without even seeing the screen. They can share answers with their colleagues in the classroom and even others outside the room.^19^ In our study we also found that 6.9% of our students accepted using mobile phones for exchange of answers during exam.

Objective structured clinical examinations (OSCEs) are widely used nowadays in the assessment of clinical skills of medical students. During OSCE most of the students share common stations and students may pass information to their colleagues.^20^ In our study, 83.6% of students accepted that they tell their friends about the questions which were asked in first shift. Nearly half of the students (42.7%) accepted that they have marked answers on the OSCE stations at some point during their course of study. Few (18.2%) also accepted that they tried to inquire answers from the teachers during OSCE.

During clinical rotations, students are observed to write fake histories and examinations which they have not asked or performed. Rennie et al^9^ reported 32% of the students accept writing neurological examination normal when it was not done. In our study 44.02% students admitted writing fake clinical histories while 28.8% admitted writing fake examination findings without actually performing it. With the changing trends in medical education there is a shift from knowledge based to competency based training and assessment. Student’s skills can be monitored by using multiple clinical evaluations during their study years like mini CEX.^21^

There may be difference in perception of academic misconduct among faculty and students. Generally students view academic misconduct less serious as compared to faculty. There is a need to bridge this gap between faculty and students perception.^22^

There were certain limitations to our study. First, it was a small sample size as compared to large number of undergraduate students and second it was a self reported study and may result in response bias.

## CONCLUSION

A large number of medical students in our institutions admitted cheating and involvement in other academic misconduct attitudes. In order to improve our educational system we need to find out the causative factors by means of larger studies and to implement strict disciplinary actions against those who are found guilty.

## Authors Contribution

KH – Conception and design, acquisition of data and final approval.

LZ – Acquisition of data, drafting and revising critically, analysis of data.

MJ – Acquisition of data, analysis and interpretation, revising critically.

SH – Designing of study, drafting, interpretation of data.
